# The Blood Pressure Pendulum following Spinal Cord Injury: Implications for Vascular Cognitive Impairment

**DOI:** 10.3390/ijms20102464

**Published:** 2019-05-18

**Authors:** Rahul Sachdeva, Tom E. Nightingale, Andrei V. Krassioukov

**Affiliations:** 1 International Collaboration on Repair Discoveries (ICORD), University of British Columbia, Vancouver, BC V5Z 1M9, Canada; sachdeva@icord.org (R.S.); tnightingale@icord.org (T.E.N.); 2 Department of Medicine, Division of Physical Medicine and Rehabilitation, University of British Columbia, Vancouver, BC V5Z 1M9, Canada; 3 GF Strong Rehabilitation Center, Vancouver Coastal Health, Vancouver, BC V5Z 2G9, Canada

**Keywords:** spinal cord injury, vascular cognitive impairment, orthostatic hypotension, autonomic dysreflexia, cerebrovascular health

## Abstract

Cognitive impairment following spinal cord injury (SCI) has received considerable attention in recent years. Among the various systemic effects of SCI that contribute towards cognitive decline in this population, cardiovascular dysfunction is arguably one of the most significant. The majority of individuals with a cervical or upper-thoracic SCI commonly experience conditions called orthostatic hypotension and autonomic dysreflexia, which are characterized by dangerous fluctuations in systemic blood pressure (BP). Herein, we review the potential impact of extreme BP lability on vascular cognitive impairment (VCI) in individuals with SCI. Albeit preliminary in the SCI population, there is convincing evidence that chronic hypotension and hypertension in able-bodied individuals results in devastating impairments in cerebrovascular health, leading to VCI. We discuss the pertinent literature, and while drawing mechanistic comparisons between able-bodied cohorts and individuals with SCI, we emphasize the need for additional research to elucidate the mechanisms of cognitive impairment specific to the SCI population. Lastly, we highlight the current and potential future therapies to manage and treat BP instability, thereby possibly mitigating VCI in the SCI population.

## 1. Introduction

It is now widely recognized that cognitive impairment is a serious consequence of SCI. The majority of spinal injuries occur in early adulthood and individuals can survive for decades with a potentially permanent impairment [1]. Cognitive functioning is a major concern not just in the rehabilitation phase, but also during re-employment and reintegration into society [2], having implications for those aging with SCI. A recent study shows that individuals with SCI are at an alarming 13-fold higher risk of cognitive impairment compared to able-bodied controls [3]. Furthermore, our recent systematic review suggests that despite substantial variability in the reported incidence, up to 60% individuals with SCI suffer from at least some degree of impairment in one or more cognitive domains, such as memory, attention, concentration, or executive function [4]. A number of studies over past four decades have unraveled various comorbid factors that are responsible for impaired cognition after SCI (reviewed in [4]). For the purpose of this minireview, we focus on major cardiovascular dysfunctions following SCI that are key contributors to vascular cognitive impairment (VCI).

**Figure 1:**
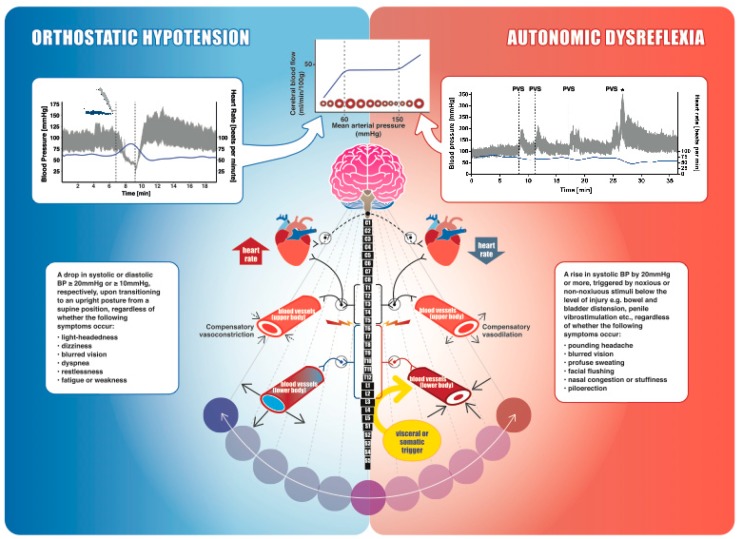
An overview of spinal cord injury (SCI), autonomic cardiovascular innervation, blood pressure (BP) instability, and cerebral autoregulation. The schematic diagram in the middle demonstrates autonomic control of the cardiovascular system. Parasympathetic control of the heart (dashed line), mediated by the vagus nerve, usually remains intact following SCI. Neurons within the brainstem provide sympathetic tonic control to spinal sympathetic preganglionic neurons. Heart and upper-body blood vessels are innervated via spinal segments T1–T5, whereas the trunk and lower extremity vasculature receive innervation from T6–L2. The splanchnic bed (liver, spleen, and intestines) is densely innervated, highly compliant, and contains approximately one-quarter of the total blood volume at rest, making it the primary capacitance bed. An SCI disrupting the sympathetic control of these vessels (i.e., at or above T6) makes them highly vulnerable to vasodilation and extreme constriction, leading to BP instability. **Orthostatic hypotension** (shown on the left): cardiovascular changes in a participant with a motor-complete cervical SCI (C5, American Spinal Injury Association Impairment Scale (AIS) A) during a head-up-tilt assessment. Beat-by-beat BP is shown in grey, and heart rate is shown in blue. BP plummeted immediately upon initiation of 60° upright tilt from the supine position and the tilt was terminated after 2 min. Mean arterial pressure was recorded as 25 mmHg at its lowest, well below the lower limit of cerebral autoregulation (top middle inset). Rebound hypertension was also apparent when the participant was returned to the supine position, further emphasizing the instability in blood pressure regulation. **Autonomic dysreflexia** (shown on the right): cardiovascular changes in a male with motor-incomplete SCI (C6, AIS C) during a sperm retrieval procedure with penile vibrostimulation (PVS), which is a visceral/somatic trigger originating below the spinal lesion. The dashed lines indicate each time the PVS is applied and is followed by significant and rapid increases in BP. * indicates ejaculation. In this case, systolic BP almost triples and mean arterial pressure is ~250 mmHg, well above the upper limit of cerebral autoregulation (top Figure). **Cerebral autoregulation curve** (shown on the top): cerebral blood flow (CBF) is shown in relation to cerebral artery lumen diameter and mean arterial pressure. The dashed lines represent the lower and upper limits of CBF autoregulation, which are exceeded by our clinical orthostatic and autonomic dysreflexia examples. Red circles represent the cerebral arteries (either vasodilating or vasoconstricting to counteract changes in systemic blood pressure), and the blue solid line represents cerebral blood flow.

Considering the segmentally differentiated autonomic innervation of the heart and vasculature, neurological level (and severity) of SCI determines the extent of subsequent cardiovascular dysfunction (Figure 1). With disruption of supraspinal sympatho-excitatory drive to spinal sympathetic preganglionic neurons (located between first thoracic and second lumbar spinal segments), the vast majority of individuals with tetraplegia or high paraplegia (SCI above T6) experience debilitating cardiovascular impairments as a result of sympathetic decentralization. In addition to a consistently low resting blood pressure (BP), decentralization of sympathetic cardiovascular control also predisposes these individuals to drastic BP fluctuations, where systolic BP can drop down to 50 mmHg or lower during an orthostatic challenge (e.g., assuming an upright posture) or can rapidly spike up to 300 mmHg as a reflex response to noxious or non-noxious stimuli originating below the spinal lesion (e.g., distended bladder). These hypotensive and hypertensive crises, called orthostatic hypotension and autonomic dysreflexia (Figure 1), are essentially the result of either insufficient or excessive vasoconstriction, respectively, and are generally present within the same individual. While a single episode of extreme BP can have dangerous consequences for the cerebrovasculature, these hypertensive and hypotensive events occur as frequently as 41 and 28 times per day, respectively [5], and thus are a significant chronic burden.

## 2. BP Lability and Cognitive Impairment after SCI: Lessons Learned from Able-Bodied Individuals

Across a wide range of systemic BPs (i.e., 60–150 mmHg mean arterial pressure), cerebral vasculature maintains a fairly uniform brain perfusion via a process called autoregulation. Furthermore, increased local metabolic demands in the brain (such as during a cognitive task) are typically coupled with an increase in regional cerebral blood flow (CBF). This phenomenon, called neurovascular coupling, ensures adequate substrate delivery and removal of metabolites within the activated brain region. Neurovascular coupling and cerebral autoregulation, along with CO_2_ vasoreactivity, maintains the spatiotemporal adequacy of cerebral perfusion [6]. Despite cerebral autoregulation, chronic hypotension in able-bodied individuals has been shown to result in cerebral hypoperfusion [7], as well as impaired neurovascular coupling [8]. More importantly, certain brain regions, e.g., the basal ganglia, hippocampus and cortex, are likely to be more susceptible to ischemic damage [7,9], resulting in significant deficits in cognitive domains such as memory, attention, and reaction time [10,11]. Similar to able-bodied hypotensive individuals, those with upper-thoracic and cervical SCI also exhibited significantly lower CBF at rest that correlated with reduced cognitive performance compared to controls. Indeed, raising systemic BP was shown to increase resting CBF, independent of the mechanism (e.g., either by an alpha-1 agonist, midodrine hydrochloride, or by a nitric oxide synthase inhibitor, nitro-L-arginine methyl ester) [12]. Interestingly, the experiments from our laboratory showed that although resting CBF was similar between SCI and age-matched controls, neurovascular coupling was significantly impaired during a cognitive task, but was improved by raising BP with the administration of midodrine hydrochloride [13], highlighting the association between low BP and impaired cognitive performance. 

Conversely, several studies have substantiated the association between chronic hypertension and cognitive decline in non-SCI individuals. Linked with impaired vascular tone, enhanced blood brain barrier permeability, and profound structural remodeling, abnormally high BP for a prolonged period of time detrimentally alters both structural and functional properties of arteries [14]. Among these aforementioned aspects, structural maladaptations in response to chronic hypertension are better understood. With higher intralumenal pressure and increased tangential stress on the artery wall, cerebral arteries become thicker as an adaptive response to protect downstream vasculature against increased BP. This, however, results in reduced lumen diameter and increased wall-to-lumen ratio, which is a major predictor for end-organ damage [15]. Consequently, in able-bodied hypertensive individuals, this results in reduced CBF at rest in cortical (occipitotemporal and prefrontal) and hippocampal regions [16]. Furthermore, regional increase in CBF during memory tasks (neurovascular coupling) is also impaired in hypertensive subjects compared to normotensive controls [17]. In individuals with SCI above T6, extreme bouts of transient hypertension (i.e., autonomic dysreflexia) are prevalent and occur numerous times per day (mean: 11 times/day) [5]. Using a rodent model, our laboratory has shown that predisposing animals to cardiovascular impairment via a high-thoracic SCI leads to deleterious structural and functional maladaptations in cerebrovasculature. Specifically, in rats with T3 SCI, the middle cerebral artery showed a significant reduction in distensibility, increased stiffness, and increased wall-to-lumen ratio [18]. This was further associated with a reduction in CBF (at rest as well as during a hypercapnic challenge) and significantly impaired short-term memory [19]. More recent work from our laboratory showed that cerebrovascular impairments are more pronounced when the rats with T3 SCI are exposed to daily repetitive autonomic dysreflexia via colorectal distension [20]. Clinical evidence has also demonstrated that the intensity of autonomic dysreflexia, quantified using a questionnaire, is inversely associated with performance in executive function tests [21]. The existing evidence linking repeated bouts of autonomic dysreflexia with cognitive impairment following SCI is still preliminary. However, given the plethora of studies supporting hypertension-related cognitive decline in able-bodied individuals, it is tempting to speculate that similar mechanisms would also underlie cognitive impairment due to transient hypertensive episodes in SCI—an avenue worthy of future research.

**Figure 2:**
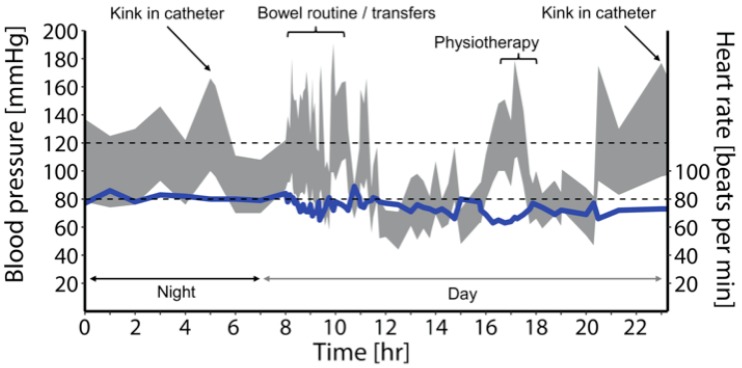
Ambulatory BP monitoring data collected from a research participant with a motor-complete cervical SCI (C5, AIS B). These data demonstrate transient, pendulum-like shifts in BP (in grey) in response to various stimuli throughout a normal day. Multiple episodes of autonomic dysreflexia (*n* = 25) and orthostatic hypotension (*n* = 33) were observed in this case, with systolic BP ranging from 71 to 180 mmHg (mean arterial pressure: 53 to 132 mmHg). Triggers for these conditions are annotated on the figure. The bowel routine in particular demonstrates aberrant BP changes, in both directions, in response to suppository insertion, digital stimulation, and pressure applied to the abdomen (autonomic dysreflexia) and transferring to and from the commode (orthostatic hypotension). Heart rate is represented by the blue solid line.

It is also noteworthy that unlike the able-bodied individuals with either persistent hypotension or hypertension, individuals with cardiovascular impairment secondary to high-level SCI generally experience both extreme ends of the BP spectrum (Figure 2). The pendulum-like swings in BP in those with a high-level SCI (i.e., during orthostatic hypotension and autonomic dysreflexia) easily reach the values beyond the autoregulatory limit [5], predisposing an individual to either ischemic or hemorrhagic stroke. A recent report from our laboratory demonstrated, in an individual with a chronic, motor-complete SCI at the T4 spinal segment, that excessive hypertension during an episode of autonomic dysreflexia (likely due to urinary tract infection) exceeded the autoregulatory limit, resulting in cortical and subcortical vasogenic edema, a condition called posterior reversible encephalopathy syndrome [22]. While discussing the upper end of Lassen’s autoregulation curve (Figure 1), it is also important to consider the lower end, beyond which vasodilation becomes ineffective and arteries tend to collapse due to low intralumenal pressure [23]. Chronic hypertension is known to increase the lower limit of autoregulation, likely via maladaptive remodeling of cerebrovasculature, and thus even less severe episodes of hypotension can be problematic [24]. It is reasonable to envision that this phenomenon may have serious implications for individuals with SCI that suffer both hypertensive and hypotensive episodes concomitantly, presenting a unique double-edged sword for this population. Adding further to the cardiovascular disease risk is the fact that owing to the amplification of various physical, physiological, and environmental risk factors, cardiovascular (and, in turn, cerebrovascular) disease progression is immensely accelerated after SCI [25]. In fact, based on our analysis of a Canadian Community Health Survey, it was found that even after controlling for risk factors such as age and sex, SCI was independently associated with a nearly 3-fold higher risk of cardiovascular disease and 4-fold higher risk of stroke [26]. This is especially concerning in light of the mounting evidence from the non-SCI population, which suggests that essentially any of the stroke etiologies can result in VCI, ranging from mild cognitive impairment to dementia [27]. 

## 3. Therapeutic Perspectives

Preventing and/or controlling volatile BP fluctuations to mitigate VCI following SCI can be approached in a number of ways. In terms of preclinical validation, this could be achieved by: (1) restoration of supraspinal control through neural regeneration [28], (2) prevention of secondary spinal cord damage through early neuroprotection [29], (3) reduction of aberrant sprouting of nociceptive afferent fibers that trigger autonomic dysreflexia episodes [30], or a logical combination of these approaches. This topic has been previously reviewed by our group [31]. From a clinical perspective, a variety of pharmacological and nonpharmacological options are available for management of autonomic dysreflexia and orthostatic hypotension that could reduce cardiovascular disease burden and decelerate the VCI trajectory following SCI [32,33,34]. A major limitation (other than the obvious side effects) of currently available pharmacotherapies is that most of the drugs are slow-acting (i.e., they take several minutes to reach effective plasma concentrations and get metabolized) and also lead to sustained, undesirable cardiovascular effects. The extreme cardiovascular events following SCI are more transient; hence, it is reasonable to question the efficacy of presently available treatments. One potential solution to this could be the employment of neuromodulation strategies such as epidural or transcutaneous spinal cord stimulation, which have demonstrated the capability to almost instantaneously modulate BP [35,36,37,38]. These studies, although promising, need further systematic exploration prior to widespread clinical implementation.

## 4. Conclusions

We are only beginning to explore the interplay between cardiovascular and cognitive impairments following SCI. Given the wealth of research in the non-SCI population, many principles can potentially be extrapolated in order to expedite our understanding of the precise mechanisms involved. Future research is necessary to develop effective strategies to prevent or ameliorate cognitive impairment in persons with SCI. Advances in these areas will significantly impact independence and quality of life in this population.
